# Osteopontin-expressing valvular interstitial cell subpopulation as a driver of extracellular matrix remodeling in aortic valve disease

**DOI:** 10.3389/fcvm.2026.1755830

**Published:** 2026-04-07

**Authors:** Răzvan D. Macarie, Monica M. Țucureanu, Letiția Ciortan, Mihai Bogdan Preda, Ileana Mânduțeanu, Elena Butoi

**Affiliations:** 1 Inflammation Department, Institute of Cellular Biology and Pathology “Nicolae Simionescu”, Bucharest, Romania; 2 Stem Cell Biology Department, Institute of Cellular Biology and Pathology “Nicolae Simionescu”, Bucharest, Romania

**Keywords:** valvular interstitial cells, aortic valve disease, cell-cell communication, extracellular matrix remodeling, bioinformatic analysis

## Abstract

**Introduction:**

Aortic valve disease (AVD) is a cardiovascular disorder highly prevalent in the elderly population. Aortic valve leaflets suffer hardening due to extracellular matrix (ECM) remodeling and subsequent calcification, leading to impaired blood flow and aortic valve stenosis. Valve interstitial cells (VICs) are fibroblast-like cells that can undergo myofibroblast activation and osteogenic transformation, contributing to disease progression.

**Methods:**

We performed a bioinformatic re-analysis of a publicly available scRNA-seq dataset to identify pathogenic VIC subpopulations and characterize cell-cell communication networks relevant to early AVD.

**Results:**

Re-analysis of scRNA-seq data from aortic valves of *Apoe*^−/−^ and *Ldlr*^−/−^ mice revealed a distinct VIC subpopulation enriched in osteopontin (*Spp1*), fibromodulin (*Fmod*), and chondrocyte-specific genes, including *Chad*, *Comp*, and *Cilp2*. Differential expression and gene ontology enrichment analyses indicated a strong signature of ECM organization and remodeling in this cluster under atherosclerotic conditions. Cell-cell communication analysis using CellChat showed enhanced intercellular signaling involving the VIC Spp1^+^ cluster. Moreover, incoming interaction strength was increased through collagen, fibronectin, Spp1, and cyclophilin A (CypA) signaling pathways, while the thrombospondin pathway was decreased. NicheNet analysis suggested a crosstalk between VICs Spp1^+^ with immune and valvular cells via receptors such as *Icam1*, *Itgav*, *osteoprotegerin*, *Sdc4*, and *Itga10*. Moreover, gene regulatory network reconstruction using pySCENIC identified NFE2L1 as a shared transcriptional regulator in both hyperlipidemic conditions, potentially driving the fibrotic program in VIC Spp1^+^ across both models.

**Discussion:**

These findings suggests the presence of a disease-associated VIC Spp1^+^ subpopulation, that may contribute to valve sclerosis through ECM remodeling and deposition, providing mechanistic insights into early valve sclerosis.

## 1 Introduction

Calcific aortic valve disease (AVD) is a prevalent and debilitating cardiovascular disorder, affecting over 25% of individuals aged 65 and more than 50% of those over 85 years of age (1), imposing a growing burden on aging populations globally. Considered for a long time a degenerative process, calcific AVD is now recognized as an actively regulated disorder involving inflammation, endothelial to mesenchymal transition, fibrosis, and osteogenic transformation of valvular interstitial cells. Despite these advances, to date, there is no approved pharmacotherapy to slow down or reverse AVD progression; the only effective treatment for advanced cases of aortic valve stenosis remains surgical replacement or transcatheter AV implantation (TAVI). While TAVI minimized the risks involved in the surgical procedure, now being recommended for high-risk patients and those over 75 (2), its high cost remains prohibitive in developing countries.

The adult aortic valve comprises three thin leaflets anchored to the annulus, covered on both surfaces by a monolayer of valve endothelial cells (VECs). Each leaflet is stratified into a collagen-dense fibrosa (providing tensile strength), a glycosaminoglycan/proteoglycan-rich spongiosa (conferring compressive damping), and an elastin-rich ventricularis with predominantly radial elastic fibers that enable rapid recoil and coaptation to sustain the repeated cardiac cycles (3). VECs sense side-specific shear (oscillatory on the fibrosa vs. pulsatile on the ventricularis) and maintain barrier and antithrombotic functions, while embedded valve interstitial cells (VICs) sustain extracellular matrix (ECM) turnover and homeostasis. Disruption of this tightly regulated VIC-ECM interaction compromises leaflet biomechanics and fosters fibrocalcific remodeling (4).

AVD progresses through two major clinical stages: *sclerosis*, characterized by inflammation, leaflet thickening, and microcalcification with preserved function, and *stenosis*, marked by ECM remodeling, extensive calcific deposition, and impaired hemodynamics (5). Histopathologically, early lesions are characterized by focal subendothelial matrix disruption and lipid accumulation beneath the fibrosa, with microcalcifications and lipid-rich cores topped by inflammatory infiltrates (6). Advanced disease features large calcific nodules, osteogenic remodeling, neovascularization, and bone-like tissue formation in the valve (7).

Progress in identifying therapeutic targets is hindered by the absence of human tissue samples from early disease stages and the lack of accurate preclinical models. While VIC cultures serve as accessible *in vitro* systems to interrogate pro-calcific signaling, VICs in 2D cultures tend to activate, inducing variability in culture conditions and thus limiting reproducibility (8). Among *in vivo* models, mice remain the most widely used. Research relies on hypercholesterolemia-induced *Ldlr^−/−^* or *Apoe^−/−^* genetic backgrounds, sometimes in combination with additional genetic alterations like Notch1, or eNOS deficiency, and exposure to high-fat diets to better mimic disease conditions (8). Although these models usually develop calcification only after prolonged induction (more than 20 weeks), they can provide valuable insights into early disease mechanisms.

Mechanistically, AVD is an active process that begins in the fibrosa layer, where VECs are exposed to circulating blood agonists such as high glucose, oxidized LDL, pro-inflammatory cytokines (TNF-α, IL-1β), or bacterial components, as well as to disturbed oscillatory shear stress. Under these conditions, VECs undergo inflammatory activation and endothelial-to-mesenchymal transition, processes driven largely by TGF-*β*, NF-*κ*B, and Wnt/*β*-catenin signaling (3, 9). Innate and adaptive immune cells (notably macrophages and T cells) amplify pathology via cytokines, inflammasome activation, and matrix-degrading enzymes.

VICs are fibroblast-like cells that act as the principal effectors in AVD pathogenesis. Quiescent VICs undergo myofibroblastic activation, a state characterized by increased *α*-smooth muscle actin (*α*-SMA) expression and the deposition of ECM, which together drive the early stages of aortic valve sclerosis. VICs can also undergo osteogenic transformation via TGF-*β*/BMP pathways, Wnt/*β*-catenin signaling, and transcription factors such as RUNX2 and MSX2, ultimately contributing to the calcific remodeling characteristic of late-stage calcific AVD (10). Experimental data support a temporal trajectory in which myofibrogenesis often precedes osteogenic commitment. In 3D cultures, VICs exposed to osteogenic stimuli first exhibit *α*-SMA up-regulation, followed by RUNX2 expression and calcific nodule formation. Conversely, disrupting myofibroblast contractility or *α*-SMA expression attenuates downstream osteogenic differentiation (10).

Single-cell transcriptomics enables the identification of novel cell states and subtypes, providing insights into the dynamic transcriptional programs that occur during cell communication and differentiation. Given the recognized heterogeneity of VIC populations, single-cell RNA sequencing (scRNA-seq) provides unique advantages for characterizing pathological cell states and their roles in disease progression. As mentioned, VIC can differentiate into myofibroblast-like cells characterized by increased *α*-SMA expression. However, some evidence suggests the existence of an intermediary matrifibrocyte-like VIC-phenotype, which may drive early subendothelial thickening, fibrotic remodeling, and overall ECM dysregulation observed in early aortic stenosis lesions (11). Defining the molecular signatures and functional properties of these distinct VIC subtypes will not only advance our understanding of AVD pathophysiology but also guide the identification of novel therapeutic targets.

The present study is based on publicly available scRNA-seq data generated by a previous investigation of aortic valves from C57BL/6J wild-type, *Ldlr^−/−^*, and *Apoe^−/−^* mice (12). They identified four main VIC subsets, *Meox1*^+^, *Id4*^+^, *Spp1*^+^, and *Irf7*^+^ positive VICs, and reported that VICs from hyperlipidemic mice displayed higher expression of genes associated with myofibroblast activation and calcification compared to normolipidemic (C57BL/6J) controls, without further subset-specific characterization.

In the current study, we performed a reanalysis of this dataset to further explore VICs' heterogeneity in AVD. Specifically, we focused on an osteopontin-positive (Spp1^+^) VIC chondrogenic subpopulation, investigated how it undergoes transcriptional reprogramming under hyperlipidemic conditions and which intercellular signals may drive its ECM-remodeling phenotype. We identified extracellular matrix organization as the dominant disease-associated transcriptional signature of this VIC subset and integrated complementary computational approaches to investigate its regulation. Ligand-receptor inference and cell-cell communication analyses highlighted candidate signaling pathways acting on VIC Spp1^+^ cells, while regulatory network analysis suggested transcriptional factors potentially controlling these ECM-related changes.

## 2 Methods

### 2.1 scRNA-Seq analysis

We used a publicly available scRNA-seq data (12), in the form of a raw UMI matrix of 6,574 cells from aortic valves of C57BL/6J (wild type), *Ldlr^−/−^*, and *Apoe^−/−^* mice. Using the R package Seurat v5 (13), the single cells that had a mitochondrial gene expression higher than 10% and a gene count less than 300 were removed. The top 30 principal components were determined by the Jackstraw method and used to perform UMAP reduction and cell clustering. Integration of the three datasets was performed using the scVI method. A clustering resolution of 0.3 was applied, resulting in 13 distinct cell clusters. To further confirm cluster identity and to resolve intra-lineage heterogeneity, we subclustered VICs at higher resolutions (0.5 and 0.8), which reproducibly identified the distinct VIC Spp1^+^ state (Supplementary Figure S1). Cell type identities were assigned based on gene expression profiles of each cluster across all conditions using Seurat's *FindConservedMarkers()* function, while also integrating the annotation provided in the original paper (Supplementary Figure S2). As an additional quality control step, hemoglobin and ribosomal protein-encoding genes were removed from the data set as their overabundance interfered with the downstream cell-cell communication analysis.

### 2.2 Differentially expressed gene (DEG) analysis

To identify genes differentially expressed between the three conditions, differential expression analysis was performed using Seurat v5. *The FindMarkers()* function was applied using the following parameters: minimum fraction (*min.pct*) of 0.1 to include only genes expressed in at least 10% of cells, a log fold-change threshold (*logfc.threshold*) of 0.25, and the non-parametric Wilcoxon rank-sum test for differential expression analysis. Wilcoxon test is a non-parametric method that does not assume normality and is widely used for exploratory single-cell RNA-seq analyses due to its robustness to zero inflation and unequal variance. *p*-values were adjusted for multiple comparisons using the Bonferroni correction, based on the total number of genes in the dataset. Genes were considered significantly differentially expressed if they had an adjusted *p-*value <0.05 and an average log fold-change (*avg_log2FC*) >1. DEGs were visualized using volcano plots generated using the EnhancedVolcano package (v 1.22) (14).

### 2.3 Functional enrichment analysis

We performed functional enrichment analysis (using the R package *clusterProfiler*, Version 4.12.6), first to identify the potential biological processes associated with the three VIC cell clusters, and subsequently to determine the functional roles of differentially expressed genes in VIC Spp1^+^ cells from *Ldlr^−/−^* and *Apoe^−/−^* mice (15). Over-representation analysis for GO Biological Processes terms was performed using the enrichGO function in *clusterProfiler*. Terms with a *p-*value <0.05 and a *q-*value <0.10 were considered significantly enriched. The results were visualized using the *dotplot()* and *barplot()* functions from the *enrichplot* package.

### 2.4 Nichenet analysis of ligand-receptor interaction

NicheNet predicts ligand-receptor interactions that might drive gene expression changes in a given target cell type by integrating prior information of intracellular signaling and transcriptional regulatory data (16). This analysis was conducted to elucidate how cell-cell communication in the aortic valve may drive the DEGs induced by the hyperlipidemia in VIC Spp1^+^. We applied a sender-focused approach, considering all aortic valve cells as potential sender cell types, and VIC Spp1^+^ as the receiver population. In this approach, all valvular cell populations were considered potential ligand sources acting on VIC Spp1^+^ cells. However, because spatial proximity cannot be inferred from scRNA-seq data, the predicted ligand-receptor interactions represent potential rather than direct signaling relationships. Potential ligands were included if their corresponding genes were expressed in >10% of cells. DEGs with an adjusted *p-*value <0.05 and an avg_log_2_FC >0.25 were used as the target list for the NicheNet analysis. Ligand activity scores were calculated using the *nichenet_seuratobj_aggregate()* and *predict_ligand_activities()* functions, integrating prior knowledge of ligand-target interactions. The top-ranked ligands were identified based on Pearson correlation coefficients between predicted and observed target gene expression. Visualization of the ligand-receptor network and ligand-target heatmaps was performed using NicheNet's *make_circos_lr()* and *make_mushroom_plot()* functions.

### 2.5 CellChat - cell-cell communication

Cell-cell communication networks were inferred using the CellChat R package (v2.1.2), applied to the single-cell RNA sequencing data from *Ctrl, Apoe^−/−^*, and *Ldlr^−/−^* mouse aortic valve samples. CellChat identifies and analyzes intercellular communication networks based on known ligand-receptor interactions (17). To avoid biases introduced by the unequal cell numbers across experimental groups (Supplementary Figure S3a), CellChat analyses were performed independently for each condition (Ctrl, *Apoe^−/−^*, and *Ldlr^−/−^*), and the resulting communication networks were compared between conditions.

Overexpressed genes and interactions were detected using *identifyOverExpressedGenes()* and *identifyOverExpressedInteractions()* functions, respectively. Communication probabilities were then computed with *computeCommunProb* (min.cells = 10), and only interactions with a default *p-*value of *p* < 0.05 were retained for downstream analysis. Network centrality measures and signaling roles of cell populations were quantified using *netAnalysis_computeCentrality()* and *netAnalysis_signalingRole_scatter()*.

Condition-specific objects were merged to compare global communication patterns across groups. Pairwise differences in the number and strength of interactions were visualized with *compareInteractions()* and *netVisual_diffInteraction()*. To visualize differential outgoing and incoming signaling associated with VIC Spp1^+^ in hyperlipidemic vs. control conditions *netAnalysis_signalingChanges_scatter()* function was used, and individual signaling ligand-receptor pairs for each signaling pathway were visualized with the *netVisual_bubble()* and *netVisual_chord()*.

To further verify that differences in cell abundance did not bias the inferred signaling programs, we performed a downsampling analysis in which control VIC Spp1^+^ cells were randomly subsampled to match the number observed in *Apoe^−/−^* mice. Re-analysis of the downsampled dataset produced highly concordant signaling patterns, supporting the robustness of the inferred communication networks (Supplementary Figure S3b).

### 2.6 Gene regulatory network inference using pySCENIC

ScRNA sequencing data from VIC Spp1^+^ were analyzed using the pySCENIC workflow (18) to infer transcriptional regulatory networks. Previously filtered expression data were imported as a Loom file into Scanpy (v1.9.8) (19), then were annotated with cell-type metadata, and only information for VIC Spp1^+^ cells was kept and analyzed. First, GRN inference with GRNBoost2 was used to identify TF–target co-expression modules; second, the regulon prediction step and context pruning were performed (*pyscenic ctx*) using motif enrichment supplied by the Aertslab (20); and third, regulon activity scoring to compute TF activity per cell was computed with AUCell. Loom outputs were imported into R, and regulon AUC matrices were extracted. Per-regulon thresholds were estimated using *AUCell_exploreThresholds*. Cells were assigned as “regulon-active” when the regulon AUC exceeded the selected threshold. Regulon activity data were processed into a binary matrix indicating the presence or absence of regulon activity, with entries set to 1 if activity was detected and 0 otherwise. Binary matrices were visualized as heatmaps using the pheatmap package (21).

### 2.7 Fluorescence microscopy

For immunofluorescence staining of murine aortic valves, hearts were embedded in optimal cutting temperature (OCT) compound, and 7 µm cryosections were prepared using a cryostat. Mouse samples included male C57BL/6 control mice, male apolipoprotein E knockout (*Apoe^−/−^*) mice, and a hyperlipidemic *Apoe^−/−^* diabetic mouse model, generated by administration of low-dose streptozotocin (STZ) to induce type 1 diabetes mellitus. These mice were obtained from our laboratory and have been previously described in a published study (22). Sections were allowed to equilibrate to room temperature, rehydrated in phosphate-buffered saline (PBS), and permeabilized with 0.1% Triton X-100 in PBS for 10 min. Non-specific binding was blocked by incubation with 3% bovine serum albumin (BSA) in PBS for 1 h at RT.

Sections were then incubated overnight at 4°C with primary antibodies diluted in PBS containing 1% BSA and 0.1% Triton X-100. The following primary antibodies were used: osteopontin (1:200; Thermo Fisher Scientific, PA5-34579) and fibromodulin (1:150; Santa Cruz Biotechnology, sc-166406).

After primary antibody incubation, sections were washed with PBS and incubated with Alexa Fluor 594-conjugated secondary antibodies (Thermo Fisher Scientific; anti-mouse A11062 and anti-rabbit A21207; 1:1000) for 1 h at room temperature. Nuclei were counterstained with 4′,6-diamidino-2-phenylindole (DAPI) for 10 min.

Sections were mounted using Fluoromount-G Mounting Medium (Invitrogen, 00-4958-02) and imaged on a Leica DMi8 inverted fluorescence microscope. Fluorophores were excited using a Spectra-X multi-LED light source (Lumencor), and images were acquired with a Leica DFC9000 sCMOS camera. Image acquisition and processing were performed using Leica LAS X software and ImageJ.

## 3 Results

### 3.1 Mouse aortic valve presents a subpopulation of interstitial cells characterized by high expression of osteopontin, fibromodulin, and chondroadherin

**Figure 1:**
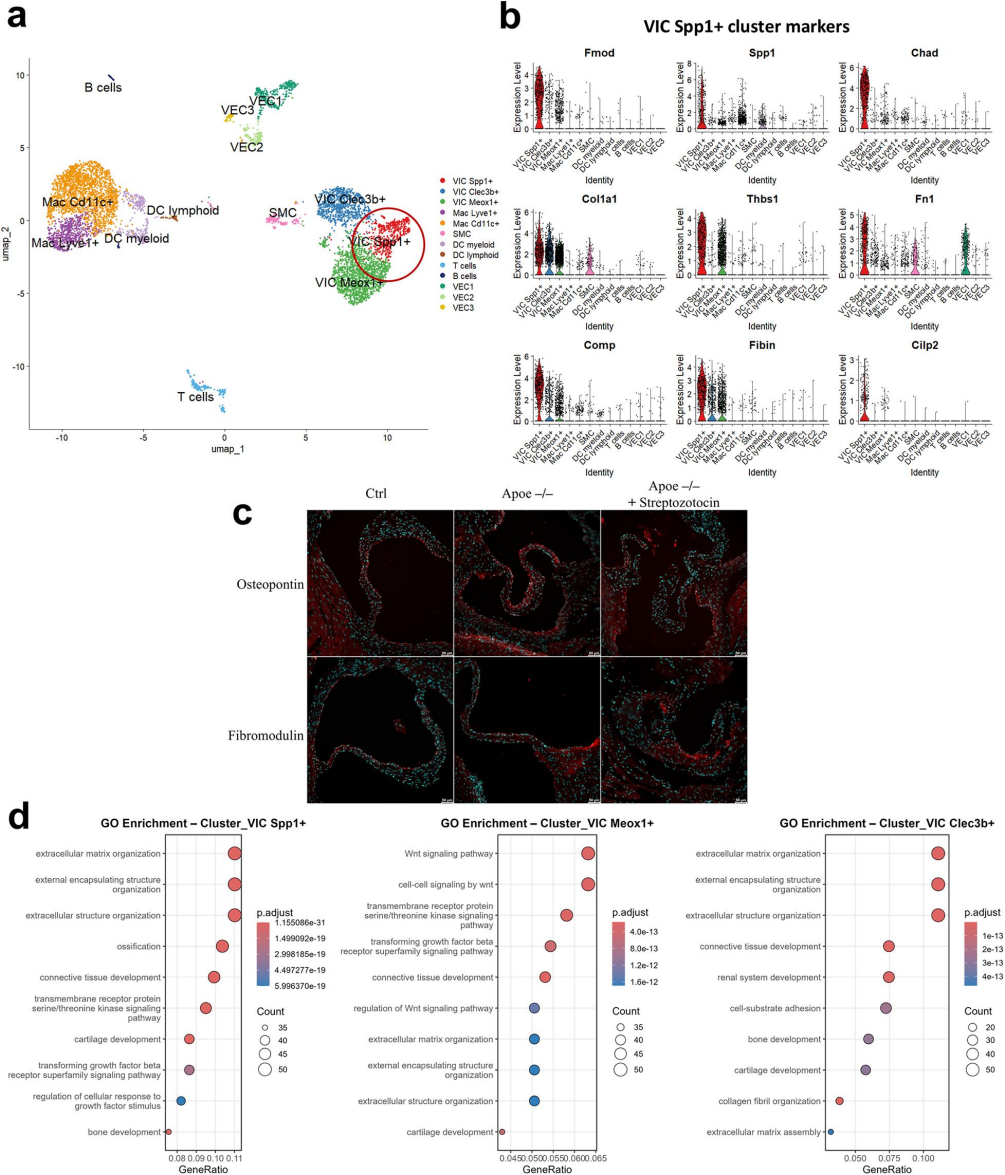
Identification of an osteopontin-expressing VIC subpopulation in murine aortic valves. **(a)**. UMAP plot of integrated single-cell transcriptomes from C57BL/6J wild-type, Ldlr^−/−^, and Apoe^−/−^ mouse aortic valves. The red circle highlights the VIC Spp1^+^ subpopulation. **(b**). Violin plots showing the expression levels of representative marker genes in the VIC Spp1^+^ cluster. **(c)**. Immunofluorescence validation of VIC Spp1^+^ associated markers in murine aortic valves. Representative sections from Ctrl -C57bl6, Apoe^−/−^ and hyperlipidemic diabetic mice showing osteopontin and fibromodulin expression (red), nuclei were counterstained with DAPI (cyan), scale bar—50 µm. **(d)**. Gene ontology (GO) enrichment of marker genes (detected in >25% of cells per cluster and with average log fold change >0.5) associated with the three VIC subpopulations. GO analysis indicates ECM structure and organization as the main biological processes in which VIC Spp1^+^ marker genes are involved.

To investigate the early mechanisms driving aortic valve dysfunction, we reanalyzed the previously published scRNA-seq dataset of aortic valves from C57BL/6J wild-type, *Ldlr^−/−^*, and *Apoe^−/−^* mice (12). Following an initial quality control and normalization, single-cell transcriptomes from all three experimental conditions were integrated and subjected to unsupervised clustering and resolved into 7 major cell populations encompassing 13 transcriptionally distinct clusters. These included two macrophage subsets, three clusters of VECs, two dendritic cell clusters, a smooth muscle cell (SMC) cluster, one B-cell and one T-cell cluster, as well as three major VIC clusters (Figure 1a). Depending on the most enriched marker genes for each of the three VIC clusters, they were named: VIC Spp1^+^, VIC Meox1^+^ and VIC Clec3b ^+^ . The marker genes defining each VIC cluster are provided in Figure 1b (VIC Spp1^+^) and in Supplementary Figure S4 (VIC Clec3b^+^, VIC Meox1^+^). Notably, the number and annotation of VIC subpopulations identified in our reanalysis differ slightly from those reported by Lee et al. (12), who described four VIC subsets (Meox1^+^, Id4^+^, Spp1^+^, and Irf7^+^). These differences are due to a more conservative clustering resolution that was used in the current study. Despite these methodological differences, the Spp1^+^ VIC population identified here corresponds closely to the VIC_C2 cluster described by Lee et al. (12), supporting the robustness of this cell state across analyses.

Gene ontology (GO) analysis was performed to determine the biological processes associated with the VIC clusters and revealed that VIC Spp1^+^ and VIC Clec3b^+^ were predominantly enriched in pathways related to ECM organization (Figure 1d). In contrast, the VIC Meox1^+^ cluster showed enrichment not only for ECM organization but also for the Wnt signaling pathway, suggesting a potentially distinct role in early pathogenic processes in the valve. Interestingly, the VIC Spp1^+^ cluster exhibits a high enrichment score for ossification (Figure 1d). Considering that this is the major pathological process that drives aortic valve disease, we further characterized this cluster. As shown in Figure 1b, the defining gene markers for VIC Spp1^+^ are mainly ECM proteins such as fibromodulin (*Fmod*), osteopontin (*Spp1*), chondroadherin (*Chad*), fibronectin (*Fn1*), cartilage oligomeric matrix protein (*Comp*), and cartilage intermediate layer protein 2 (*Cilp2*). *Spp1* encodes a multifunctional 44-kDa acidic glycoprotein involved in biomineralization and ossification. To further support the presence of the VIC Spp1^+^ cluster *in vivo*, we performed histological validation in two independent murine models of valvular disease: male *Apoe^−/−^* mice and hyperlipidemic diabetic *Apoe^−/−^* mice induced by low-dose streptozotocin. Immunostaining revealed that both Osteopontin and Fibromodulin were highly expressed in diseased valves (Figure 1c), with a preferential localization in the hinge region, an area prone to lipid accumulation. Importantly, the detection of these markers across both models provides additional evidence for the robustness of our scRNA-seq findings and supports the relevance of this VIC population in valvular pathology. Notably, while osteopontin expression is minimal or absent in normal aortic valves, it is markedly upregulated in calcified aortic valves, supporting its established role as a biomarker for assessing calcification severity (23).

Fmod is secreted by several cell types, including keratinocytes, fibroblasts, and chondrocytes, underscoring its broad role in ECM organization. It regulates collagen cross-linking by directly interacting with both collagen fibers and lysyl oxidase (24), a process essential for post-natal development and structural maturation of the aortic valve (25). Similarly, *Chad*, *Comp*, and *Cilp2* are genes typically expressed by chondrocytes, with Chad and Comp proteins known to contain collagen-binding domains, thereby acting as mediators between cells and ECM. The elevated expression of these genes suggests that this Spp1^+^ VIC cluster may represent a matrifibrocyte-like fibroblast population, specialized in ECM maintenance and remodeling.

To further contextualize the VIC Spp1^+^ population and assess the potential of EndMT contribution, we performed trajectory inference using Monocle3 on VICs and VECs while preserving the scVI-integrated manifold from Seurat analysis. VECs and VICs followed distinct trajectories (Supplementary Figure S5c), and canonical endothelial markers (Pecam1, Cdh5, Tek, Vwf) did not show a graded expression across VIC states, providing no conclusive evidence for a broad EndMT transition in this dataset (Supplementary Figure S5d). However, the Pecam1 and Tek were found to be expressed in VIC Clec3b^+^ and VIC Meox1^+^. VIC-restricted pseudotime analysis revealed an organization of VIC states along the inferred graph, with enrichment toward the VIC Spp1^+^ region depending on root selection, which we interpret as relative ordering rather than definitive lineage directionality (Supplementary Figure S5a). Consistently, ECM-associated genes (Fn1, Fmod, Spp1, Vim) displayed structured expression along the trajectory (Supplementary Figure S5b), supporting the view that VIC Spp1^+^ cells represent a distinct activation state linked to matrix remodeling.

### 3.2 Genes differentially expressed in VIC Spp1^+^ are mainly involved in ECM organization, ECM structure, and collagen fibril formation in both apoe^−/−^ and LDLR^−/−^ mice

**Figure 2:**
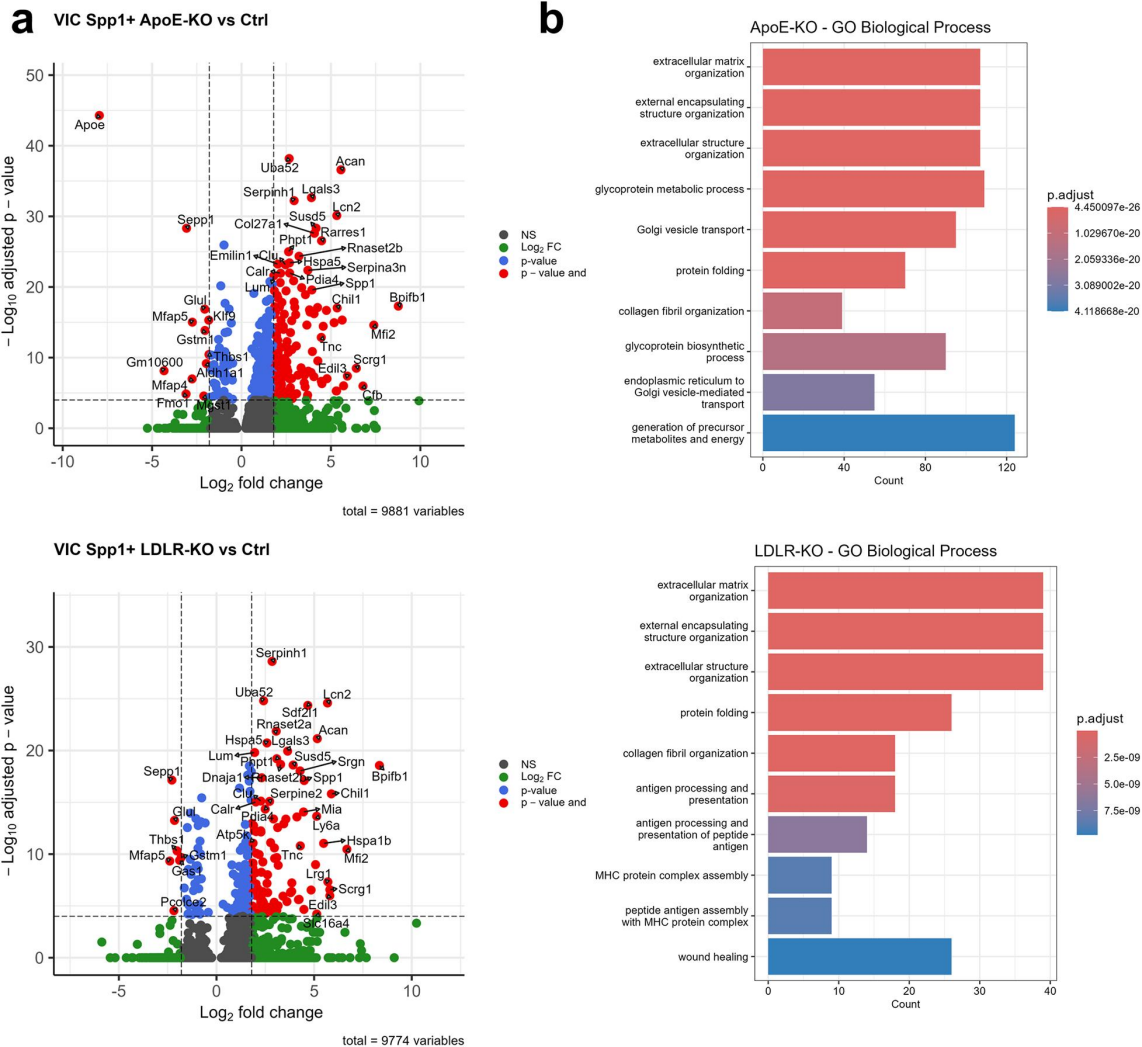
Differentially expressed genes (DEGs) in VIC Spp1^+^ cells reveal enrichment in extracellular matrix organization and collagen fibril formation in ldlr^−/−^ and apoe^−/−^ mice. **(a)**. Volcano plot showing differentially expressed genes (DEGs) between Ldlr^−/−^, and Apoe^−/−^, and control mice. **(b)**. GO enrichment analysis of DEGs in VIC Spp1^+^ cells indicate predominant involvement in extracellular matrix (ECM) organization, ECM structural components, and collagen fibril formation in both hyperlipidemic conditions.

Differential gene expression analysis comparing the VIC Spp1^+^ derived from hyperlipidemic *Ldlr^−/−^* and *Apoe^−/−^* mice vs. C57BL/6J control revealed that there is a higher expression of *Spp1*, *Chil1*, *Serpinh1* (a collagen chaperon), and of proteoglycans *Acan* and *Tnc* (Figure 2a) genes, in both hyperlipidemic conditions. Thrombospondin1 (*Thbs1*), *Sepp1*, *Mfap4*, and *Mfap5*, known to be involved in ECM remodeling processes, were down-regulated in VIC Spp1^+^ in both pathologic conditions, when compared to control. Between these genes, *Mfap4* and *Mfap5* encode extracellular glycoproteins that are involved in microfibril assembly and elastogenesis (26). Although the role of Thbs1 in cardiovascular pathologies is context-dependent, reduced Thbs1 expression has been linked to increased local fibrosis, potentially by an attenuated inhibitory effect on Timp1 (27).

Analysis of the main biological processes associated with these DEGs revealed that under atherosclerotic conditions, VIC Spp1^+^ cluster is predominantly enriched for GO terms related to ECM structure, ECM organization, and collagen fibril formation (Figure 2b), suggesting an overall dysregulation in ECM remodeling. In contrast, the other two VIC clusters displayed enrichment primarily for endoplasmic reticulum stress response (Supplementary Figure S4), supporting the idea that VIC Spp1^+^ subset constitutes the principal VIC phenotype driving ECM remodeling in hyperlipidemic conditions.

### 3.3 ICAM1, TNFRSF11B, and SDC4 expressed on VIC Spp1^+^ may mediate intercellular signaling and modulating DEGs in hyperlipidemic conditions

Cell-cell communication plays a central role in maintaining valvular homeostasis by coordinating signaling between endothelial, interstitial, and immune cells. Perturbations in these intercellular interactions can accelerate inflammatory activation, maladaptive ECM remodeling, and ultimately valvular pathology. In order to understand how intercellular communication in the aortic valve might induce the observed changes in VIC Spp1^+^, we employed NicheNet, a ligand-receptor-target inference framework that prioritizes ligands most likely to drive a specified transcriptional program. DEGs identified in VIC Spp1^+^ from both hyperlipidemic conditions (*Ldlr^−/−^* and *Apoe^−/−^*) were used separately as targets for the NicheNet ligand-receptor interaction analysis and ligands from all the valvular cells were considered as potential sender cells.

**Figure 3:**
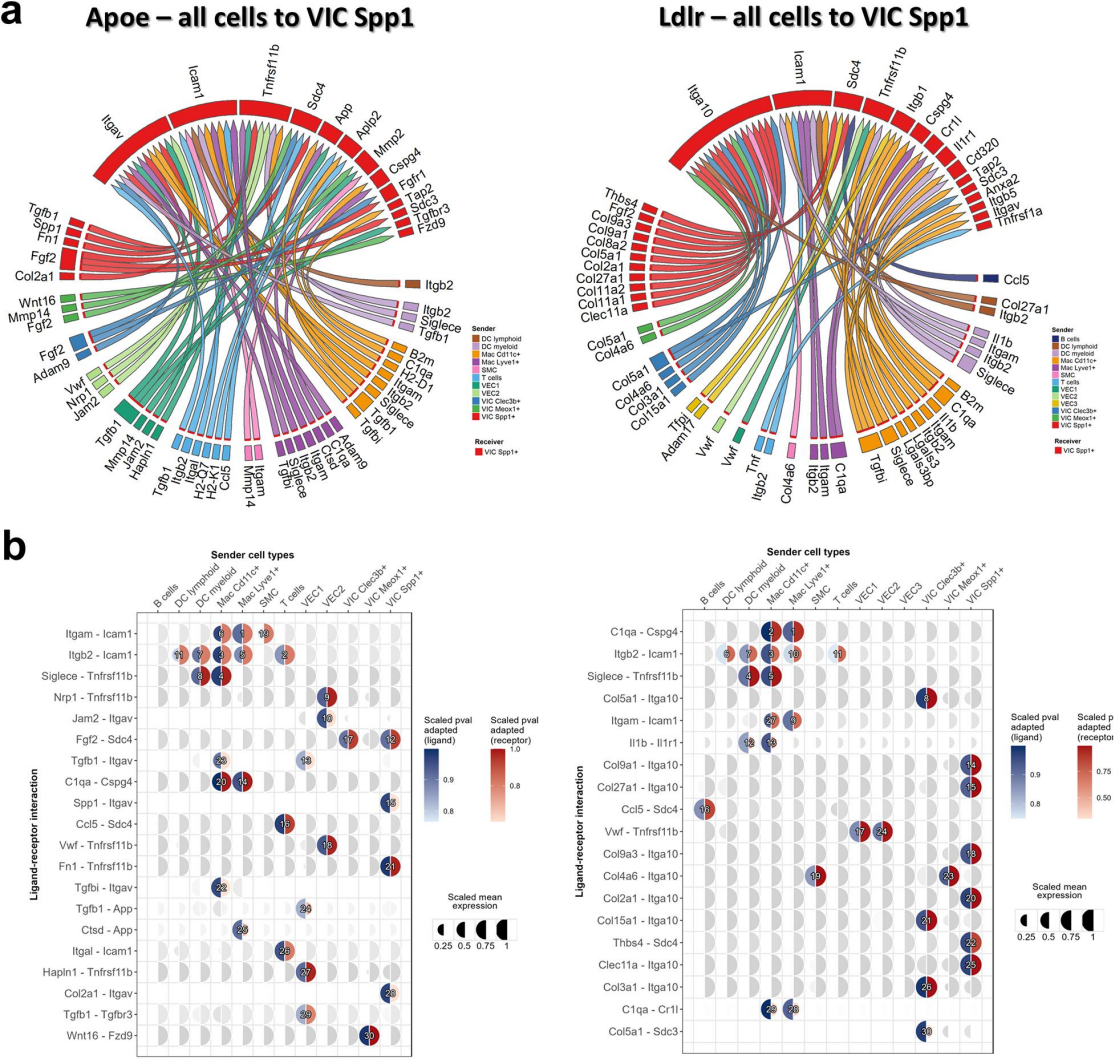
NicheNet ligand-receptor-target inference analysis prioritizing ligand-receptors predicted to drive DEGs identified in VIC Spp1^+^ cells from hyperlipidemic mice (ldlr^−/−^ and apoe^−/−^). **(a)**. Chord diagram illustrating ligands expressed by sender cells (all valvular cell types) and their corresponding receptors on VIC Spp1^+^ that may influence target DEGs under hyperlipidemic conditions. **(b)**. Mushroom plots showing cell type–specific ligand–receptor interactions with VIC Spp1^+^ cells designated as receivers. Each semicircle represents either ligand (blue) or receptor (red) expression across clusters. Circle size indicates the percentage of cells expressing the gene, while color intensity reflects scaled mean expression (0–1).

The analysis predicted a strong autocrine regulation potential within VIC Spp1^+^, mainly by Fgf2 signaling, as well as by collagen binding to Itga10 in *Ldlr^−/−^* mice, and to Itgav in *Apoe^−/−^*mice (Figure 3a). In both pathological conditions, Icam1, Tnfrsf11b, Sdc4, and Sdc3 were predicted as top-ranked receptors involved in cellular communication. The involvement of Icam1 and Tnfrsf11b (osteoprotegerin) suggests that VIC Spp1^+^ has the potential to modulate both pro-inflammatory and osteogenic stimuli. The significance of these ligand-receptor pairs is further highlighted in the mushroom plot in Figure 3b, which shows the upregulation of Icam1 and Tnfrsf11b receptors on VIC Spp1^+^ under both atherogenic conditions. Among the top-ranked interactions, Itgb2-Icam1 and Siglece-Tnfrsf11b were consistently prioritized and displayed strong expression in hyperlipidemic valves, indicating potential direct communication between VIC Spp1^+^ cells and myeloid populations. In particular, CD11c^+^ macrophages and other myeloid cell types expressed Itgb2 and Siglece, positioning them as likely sources of these signals. In addition, the *α*M integrin subunit Itgam (CD11b), which forms the Mac-1 complement receptor together with Itgb2 and is expressed by both macrophage subsets and myeloid dendritic cells, was also identified by NicheNet as a potential ligand for Icam1 on VIC Spp1^+^ cells.

In addition to VIC-macrophage interaction, the NicheNet analysis also identified a VIC-VEC crosstalk mediated by von Willebrand factor (vWF) and osteoprotegerin in the hyperlipidemic mouse model, interaction that may reflect endothelial participation in the valve microenvironment under hyperlipidemic conditions.

### 3.4 VIC Spp1^+^ showed enhanced intercellular communication potential under atherogenic conditions, mediated by collagen and fibronectin signaling pathways

**Figure 4:**
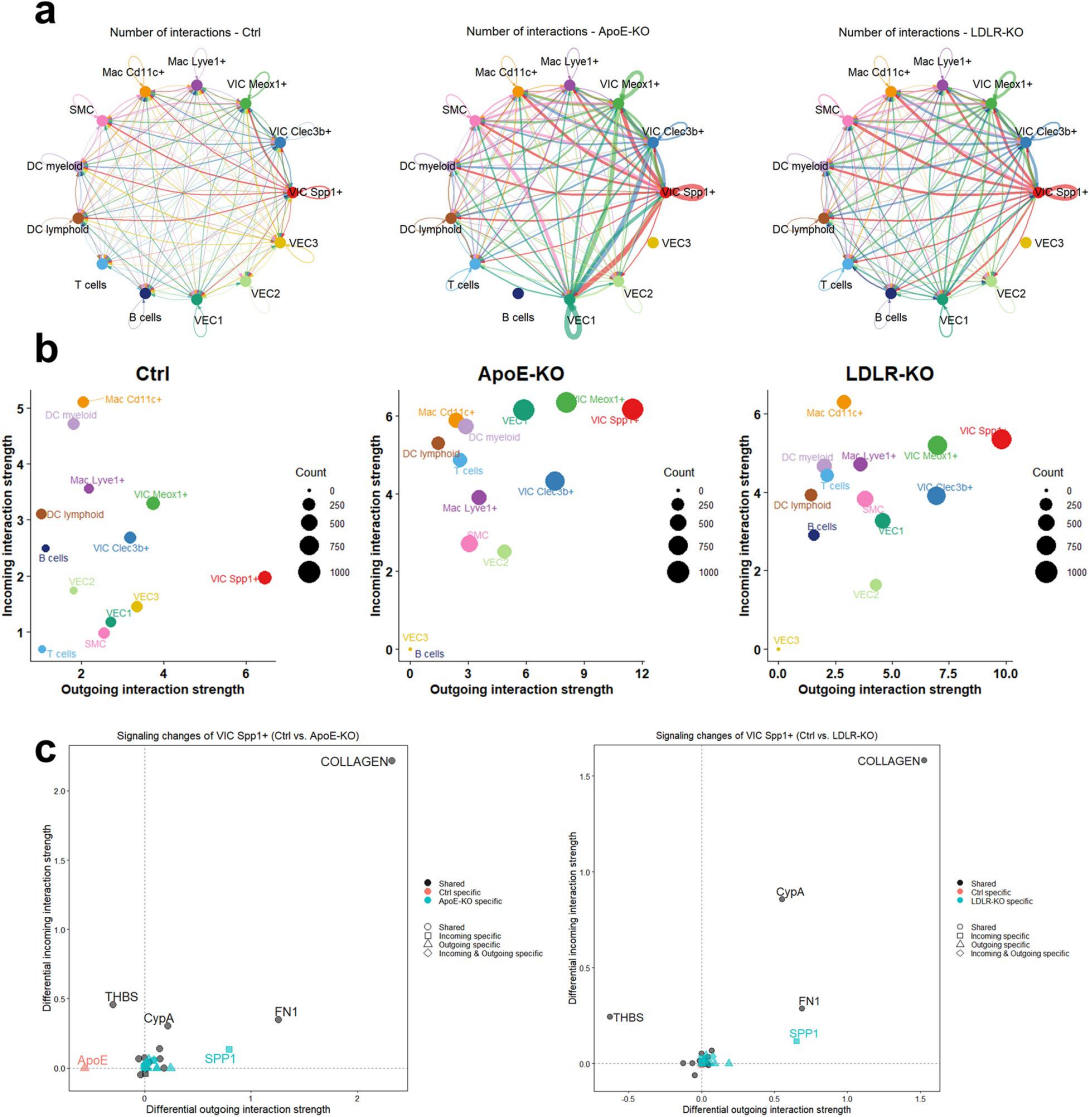
Cellchat intercellular communication analysis of VIC Spp1^+^ interactions. **(a)**. Circle plot showing the number of ligand-receptor interactions between cell types. The width of the edges corresponds to the total number of interactions. VIC Spp1^+^ cells exhibit an increased number of total interactions under pathological (Apoe^−/−^ and Ldlr^−/−^) conditions compared with control. **(b)**. Dot plot depicting the total interaction strength across control, Apoe^−/−^, and Ldlr^−/−^ groups. Dot size correlates with the number of interactions. **(c)**. Visualization of differential outgoing and incoming signaling pathways reveals increased signaling activity in collagen, Fn1, Spp1, and Cypa1 pathways, and decreased signaling via the THBS1 pathway.

To delineate the contribution of VIC Spp1^+^ to the communication networks within the aortic valve, we employed CellChat, which enables a systematic and comparative analysis of cell-cell communication. This analysis revealed that the cellular interaction network of the aortic valve is complex, and the total number of interactions coming to and from VIC Spp1^+^ is highly increased in hyperlipidemia (Figure 4a). Notably, the autocrine signaling predicted by NicheNet (Figure 3) was further confirmed by CellChat, reinforcing the potential role of this cluster in driving the pathological processes. As shown in Figure 4b, the strength of outgoing and incoming interactions is stronger in both atherogenic conditions. VIC Spp1^+^ cluster displayed the highest outgoing interaction strength of all other cell types, suggesting that this cluster is a highly interactive cell type that can be activated in pathological conditions.

To identify pathway-level alterations specific to *Ldlr^−/−^* or *Apoe^−/−^,* we compared the signaling networks coming to or from VIC Spp1^+^, and found that the strength of collagens signaling, followed by Fn1, CypA, and osteopontin pathways is increased, while Thbs signaling is decreased (Figure 4c). Since Thbs-1/2 is an important activator of TGF-*β*1 (28), the marked decrease in Thbs-1/2 signaling observed under both hyperlipidemic conditions may lead to impaired pro-fibrotic signaling, potentially acting as a compensatory mechanism.

Looking at individual ligand-receptor pairs in each of these altered signaling pathways, we observed that osteopontin secreted by VIC Spp1^+^ cells can interact with Cd44 expressed on VIC Meox^+^, SMC, Mac Cd11c^+^, and dendritic cells (Supplementary Figure S6a). These observations are supported by previous data showing that osteopontin is strongly upregulated in calcified human valves where it can interact with Cd44 and *α*v-integrins to modulate calcium deposition (29). In addition, correlations between osteopontin expression and specific immune cell populations, including dendritic cells, were reported in calcific aortic valve disease (30).

**Figure 5:**
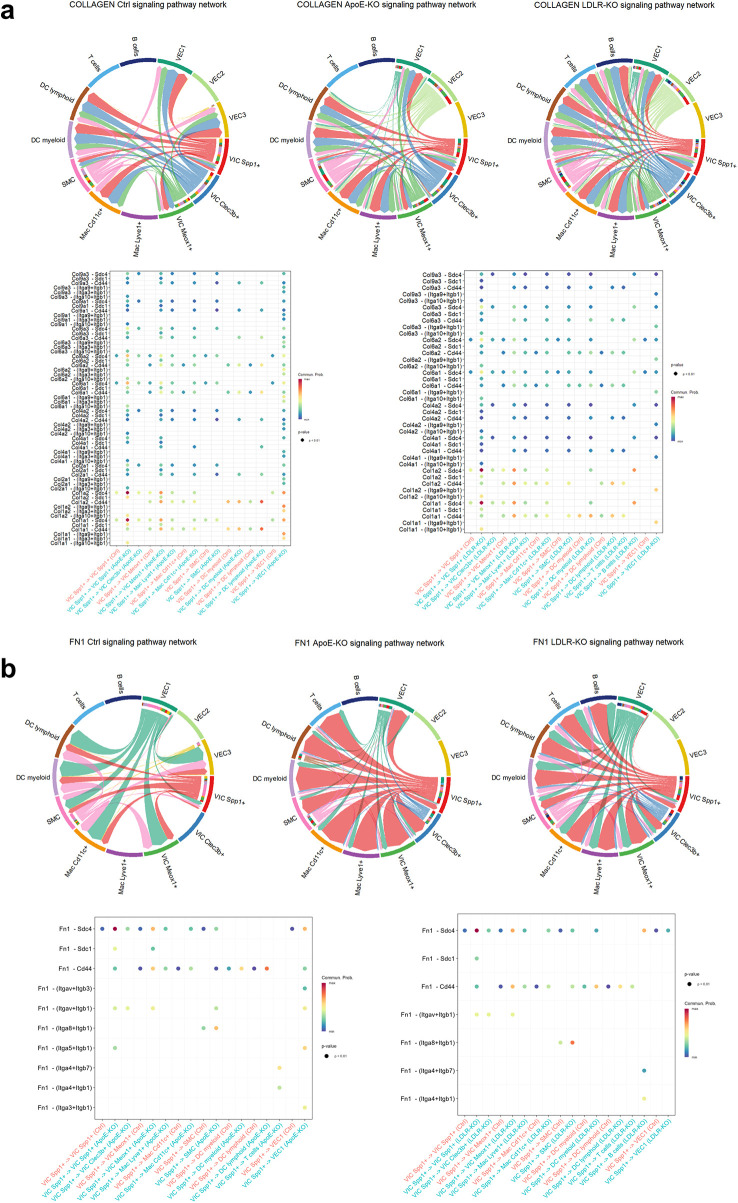
Cell-cell communication mediated by collagen and fibronectin signaling pathways is increased under pathological conditions. **(a)**. Chord plot illustrates the directionality of the collagen signaling pathway, highlighting interactions between sender and receiver populations. Dot plots show the predicted communication probabilities for all ligand-receptor pairs, with the strongest collagen-mediated signaling observed through integrin receptors Itga3b1, Itga9b1, and Itga10b1, as well as Sdc4, Sdc1, and Cd44. **(b)**. Fibronectin (Fn1) signaling was also predicted to occur primarily via Cd44 and Sdc4 receptors. In both plots, the dot color indicates communication probability, and the dot size represents *p*-values derived from a one-sided permutation test. Empty spaces denote zero communication probability.

Regarding cell-cell communication mediated by collagen and fibronectin signaling pathways (Figure 5), Cellchat analysis predicted that the highest communication probability for collagen occurs through the collagen binding integrins Int-α3*β*1, *α*9*β*1, and *α*10*β*1, but also through Sdc4, Sdc1, and Cd44 (Figure 5a). Where Cd44 showed expression on macrophages and dendritic cells, Sdc4 was expressed by VICs and the VEC1 subtype. This finding is consistent with the NicheNet analysis (Figure 3a), which highlighted that collagen signaling strongly modulates VIC Spp1^+^ activity, including through a pronounced autocrine effect, as this cluster exhibits high collagen expression. Furthermore, Fn1 signaling also shows an increase in both pathological conditions, and VIC Spp1^+^ was identified as an important source of fibronectin (Figure 1c). Fn1-mediated communication was predicted to also occur through Cd44 and Sdc4 receptors, expressed by VIC Spp1^+^, VIC Meox1^+^, but also by VEC1 cluster (Figure 5b).

### 3.5 NFE2L1 regulon activity is increased in VIC Spp1^+^ under hyperlipidemia

**Figure 6:**
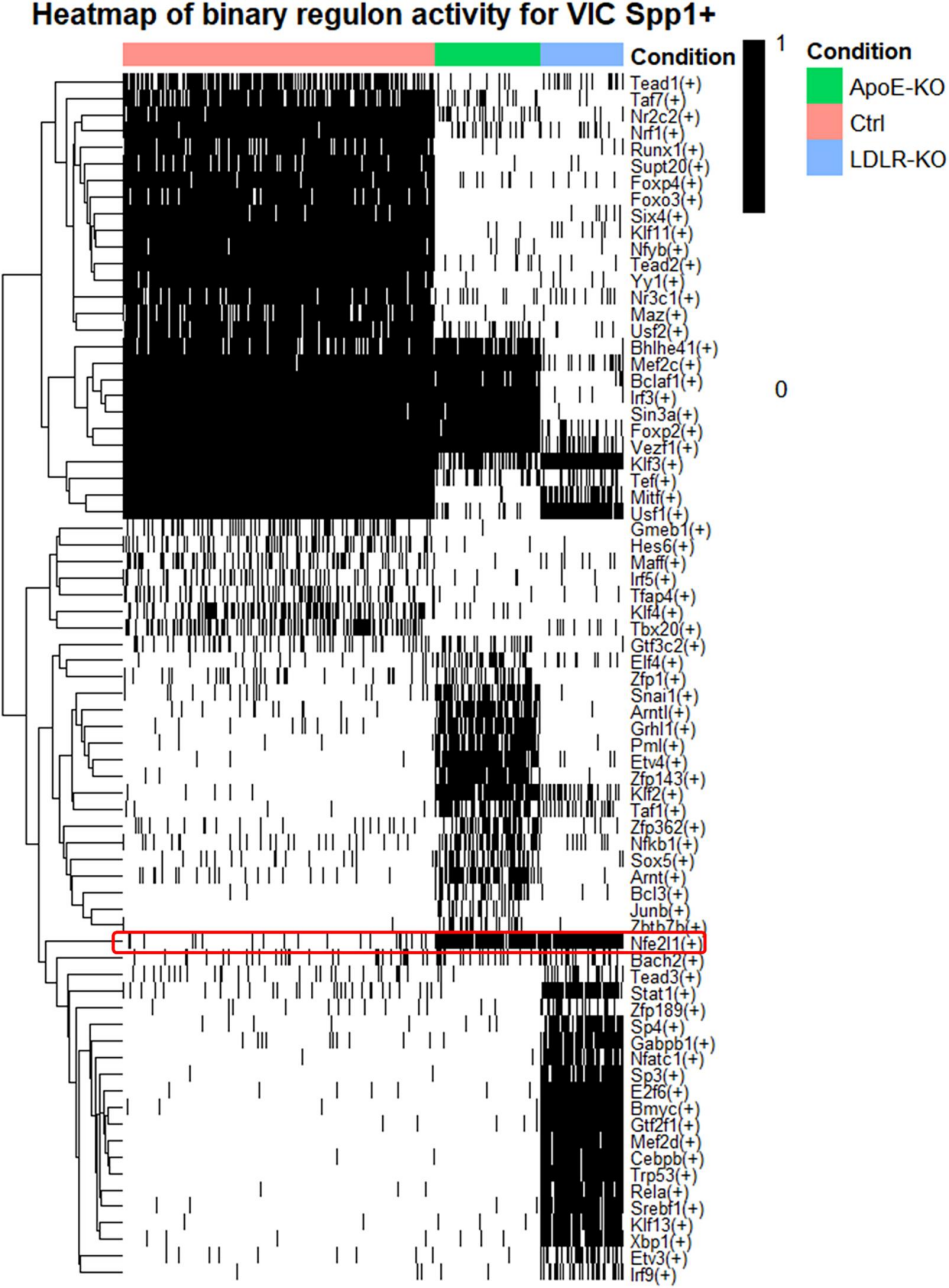
NFE2L1 regulon is activated in VIC Spp1^+^ under hyperlipidemia. Binary heatmap of transcription-factor regulon activity in VIC Spp1^+^ cells from C57BL/6J (orange), Ldlr^−/−^ (blue), and Apoe^−/−^ (green) mice, inferred using pySCENIC. cells are marked as active (1) or inactive (0) for each regulon. Hyperlipidemic models show distinct TF activation patterns, with NFE2L1 consistently active in both Ldlr^−/−^ and Apoe^−/−^ (red highlight).

Finally, using pySCENIC pipeline, we assessed the activation level of transcription-factor regulons (i.e., genes controlled by a given transcription factor). When analyzing the regulons altered specifically in the VIC Spp1^+^ cluster (Figure 6), we observed distinct activation patterns under hyperlipidemic conditions: some transcription factors were uniquely activated in *Ldlr^−/−^* mice (STAT1, MYC (Bmyc), GTF2F1, MEF2D, CEBPV, RELA), others only in *Apoe^−/−^* mice (SNAI1, ARNTL, ETV4, PML, ZFP143), and a third group only in C57BL/6J controls (FOXO3, FOXP4, NRF1, MAZ). Even if both knock-out models share hyperlipidemia as a driver, they engage distinct upstream networks, the heterogeneity of which may reflect in differences such as lipoprotein handling, inflammation, or oxidative stress.

However, the NFE2L1 regulon was consistently activated in both hyperlipidemic models (Figure 6). NFE2L1 (also known as NRF1/TCF11) is a redox-sensitive bZIP transcription factor that orchestrates the proteasome “bounce-back” response. Under conditions of proteotoxic stress, NFE2L1 undergoes proteolytic processing, translocates to the nucleus, and activates the expression of proteasome subunits and associated stress-adaptation genes via ARE-like regulatory elements (31).

Beyond its canonical role in proteasome regulation, NFE2L1 has also been identified as a direct cholesterol sensor that binds to and specifically senses cholesterol within the ER membrane (32). This dual role positions NFE2L1 as a key adaptive regulator of both proteotoxic and lipid-driven stress. The consistent activation of the Nfe2l1 regulon in *Ldlr^−/−^* and *Apoe^−/−^*mice likely reflects an integrated stress-adaptation response to proteostasis imbalance and excess cholesterol, both of which are central features of hyperlipidemia-induced valve dysfunction.

## 4 Discussion

Through a bioinformatic approach, the present study characterized a distinct chondrocyte-like VIC subpopulation, defined by high expression of Spp1 and ECM-related genes, including *Comp/Chad/Cilp2/Fmod*. This subpopulation appears to have a role in ECM remodeling under hyperlipidemic conditions, potentially contributing to the early pathological remodeling observed in AVD.

Osteopontin is a multifunctional phosphoprotein encoded by the Spp1 gene, involved in a wide range of biological processes in a tissue- and context-specific manner (33). It is present in aortic valvular lesions and has been associated with valvular calcification (34, 35). Previously, we observed elevated osteopontin levels within the aortic valve leaflets of diabetic *Apoe^−/−^*mice, further supporting its active role in the progression of aortic valve pathology (22). In patients, osteopontin was found to be increased in the pre-calcification stages of aortic valve degeneration and was associated with macrophage infiltration and calcium aggregation in patients affected by aortic stenosis or regurgitation (36). In addition, plasma levels of osteopontin from patients correlate positively with the severity of aortic valve calcification (37). However, osteopontin may not act solely as a pro-calcification factor, since there is evidence showing that osteopontin can prevent BMP4-mediated biomineralization through functional interaction with CD44v6. Thus, disruption of CD44-osteopontin functional interaction by blocking osteopontin, CD44, or their downstream signaling pathway pAkt resulted in calcium deposition *in vitro* (29).

In addition to osteopontin, our analysis showed that the VIC Spp1^+^ subpopulation expresses a group of cartilage- and matrix-associated adaptor proteins (*Comp/Chad/Cilp2/Fmod*), which bridge collagens to cell-surface receptors and regulate fibril spacing and cross-linking. These structural features allow VIC Spp1^+^ to influence leaflet biomechanics even before overt calcification becomes apparent. Notably, developmental studies indicate striking similarities between molecular mechanisms that control heart valve cell differentiation and those regulating cartilage formation (38). Among these matrix proteins, Fmod can influence collagen cross-linking and anchoring-domain organization within the valvular ECM, while Comp/Chad/Cilp2 can provide collagen-binding interfaces. Previous proteomic analysis of fibrotic vs. non-diseased aortic valve leaflets showed that Comp is among the most overrepresented proteins in the fibrotic stage (11). Although its precise role in aortic valve remodeling is not known, studies in skin suggest that Comp plays a critical role in the structural organization of the ECM by modulating type 1 collagen synthesis and fibrillogenesis (39). In dermal fibroblasts, once the fibrotic process is initiated, TGF-*β*1 upregulates Comp expression, followed by a self-perpetuating feedback loop in which Comp also enhances TGF-*β* activity (40). By analogy, these observations suggest a plausible Comp-TGF-*β*1 axis that may contribute to valvular matrix remodeling, warranting targeted investigation in the context of the aortic valve.

A similar VIC subpopulation named matrifibrocyte-like, characterized by a high expression of Comp, Fmod, Chad, and Cilp2, was found at post-natal day 30, predominantly in the hinge region of aortic valves, but not in the mitral valves (41). Consistent with this, it was also shown that the calcification-prone fibrosa side of the aortic valve from human AVD patients exhibits increased abundance of Comp, Fmod, Chad, and Cilp compared with the ventricularis or spongiosa layers (11). This chondrocyte signature of VIC Spp1^+^ maps onto early fibrosis in human AVD and aligns with the fibrosa-side predominance of ECM reorganization (42). Our analysis also revealed that the VIC Spp1^+^ cluster displayed preferential upregulation of gene programs involved in ECM and collagen fibril organization in both hyperlipidemic conditions (*Ldlr^−/−^* and *Apoe^−/−^*), in contrast to the other two VIC clusters, which exhibited a dysregulation of genes involved in ER stress response. Despite distinct genetic and mechanistic differences between *Ldlr^−/−^* and *Apoe^−/−^* mice, both models converge on chronic hyperlipidemia, which likely drives the observed valve phenotypes and may underlie the similar transcriptional and intercellular communication changes detected across both pathological conditions.

To further elucidate the role of VIC Spp1^+^ within the valvular microenvironment, we examined their putative communication with the other resident and immune cell populations. Cellular communication represents a fundamental mechanism by which multicellular systems adapt to environmental fluctuations and maintain tissue homeostasis through the exchange of biochemical and biomechanical cues. In the aortic valve, complex interactions between VEC, VIC, and immune cells orchestrate leaflet remodeling and disease progression.

NicheNet analysis indicated that transcriptional changes in both hyperlipidemic conditions can be modulated through ligand-receptor interactions involving receptors expressed on VIC Spp1^+^, including ICAM1, SDC4, TNFRSF11B, and integrins *β*1, *α*v, and *α*10. Moreover, in *Ldlr^−/−^* mice, a high degree of VIC Spp1^+^ autocrine signaling occurs through the binding of various collagens to Itga10 (Figure 5a). Previous studies showed that *α*10*β*1 integrin heterodimer is the most abundant collagen-binding integrin in cartilaginous tissues, it functions as a marker for chondrocyte differentiation, and mediates cell-ECM interactions essential for cartilage development (43). SDC4 exerts multifaceted effects on ECM remodeling. Therefore, its extracellular domain interacts with collagen fibrils to promote cross-linking (44), and binds concurrently to osteopontin, protecting it from thrombin-mediated proteolytic cleavage—a process that reduces collagen synthesis (45). The prioritization of ICAM1 and TNFRSF11B (osteoprotegerin) as important receptors on VIC Spp1^+^ by the NicheNet analysis suggests that this population can modulate both osteogenic signals, as osteoprotegerin is known to inhibit aortic valve calcification (46), and pro-inflammatory cues mediated by ICAM1. ITGB2-ICAM1 axis prioritized by the NicheNet analysis is consistent with enhanced immune engagement involving dendritic cells, macrophages, and T cells. In other tissues, such immune-fibroblast interactions contribute to extracellular matrix remodeling through cytokine release and matrix-modifying enzyme production (47). Also, LFA-1-mediated ICAM-1 signaling has been shown to activate pro-osteogenic signaling programs in aortic VICs, an effect that is further amplified under inflammatory conditions (48).

CellChat analysis identified the VIC Spp1^+^ as a highly interactive cell population displaying an increased potential for interaction within the aortic valve. Under hyperlipidemic conditions, both the number and strength of inferred interactions were markedly elevated. The signaling pathways with the highest interaction strength were collagens, fibronectin 1, and osteopontin pathways, while thrombospondin-mediated signaling was decreased. Given the high abundance of ECM components such as fibronectin and collagens within VIC Spp1^+^, it is likely that their major influence on other valvular cell types occurs through ECM modulation, a key driver of valvular disease progression. In fact, in human stenotic valves, increased deposition of type I and III collagens and profound ECM reorganization are well-documented hallmarks of disease progression. Collagen accumulation and matrix stiffening amplify VIC activation through collagen-integrin axes, such as *α*2*β*1 and *β*1, promoting a pro-fibrotic phenotype (49, 50). Likewise, fibronectin accumulates in the fibrosa during the sclerosis phase, where it regulates collagen fibrillogenesis and serves as a critical bridge between cells and the ECM through its integrin- and proteoglycan-binding domains (51). Enhanced fibronectin-integrin signaling could therefore reinforce stress fiber formation, migration, and acquisition of a matrifibrocyte-like phenotype (52), further linking VIC Spp1^+^ activation to early matrix remodeling and fibrosis in AVD.

Interestingly, all three signaling pathways predicted to be enhanced under hyperlipidemia share a common mediator—syndecan 4 which functions both as a mechanotransducer and as an ECM receptor (53). Syndecan 4 is a transmembrane heparan sulphate proteoglycan with a critical role in focal adhesion formation, in cooperation with integrins (54). It is also essential for an optimal fibroblast response to the fibrin-fibronectin provisional matrix deposited during tissue injury (55). Loss of Sdc4 gene in mice delays wound healing due to impaired fibroblast motility. However, studies addressing its role in AVD are lacking. Given that Sdc4-dependent communication was also predicted by the NicheNet analysis, its potential contribution to abnormal ECM remodeling in aortic stenosis could warrant further investigation.

Finally, employing pySCENIC analysis of the VIC Spp1^+^ cluster, we characterized distinct regulon activation profiles and identified a robust activation of the NFE2L1 regulon across both hyperlipidemic mouse models. NFE2L1, also known as Nrf1, is a highly conserved transcription factor that belongs to the CNC-bZIP subfamily and plays a central role in the proteotoxic stress and oxidative stress cell response (31), well-documented drivers of VIC osteogenic reprogramming and early leaflet remodeling in calcific AVD (56, 57). Importantly, pharmacological mitigation of ER stress has been shown to reduce VIC calcification (58), further supporting the pathogenic relevance of this pathway. The consistent engagement of NFE2L1 in hyperlipidemic VICs suggests that elevated intracellular cholesterol and proteostasis burden converge on this regulatory hub during early aortic valve disease. While our analysis highlights NFE2L1 as a promising candidate regulator of lipid-induced VIC Spp1^+^ phenotype, evidence for its role in human AVD, functional manipulation in VIC/VEC models, or genetic association analyses remains to be established. As such, NFE2L1 remains a putative regulatory candidate, identified through predictive computational inference.

In conclusion, the identification of a chondrocyte-like VIC subpopulation as an intermediate phenotype, before pathological osteoblasts, provides novel insights into the early mechanisms of AVD. This intermediate cell type may act as a pivotal mediator of ECM remodeling and thus represents a potential therapeutic target to prevent the progression from initial subendothelial thickening to overt calcification. However, future computational and experimental perturbation studies are needed to further validate the regulatory roles of this VIC subpopulation in ECM remodeling and osteogenesis.

This study, however, has several limitations. First, the results derive exclusively from *in silico* analyses and should be viewed as mechanistic hypotheses. Functional validation *in vitro* or *in vivo* is necessary to establish causality. Second, the analyses are restricted to murine hyperlipidemic models (*Ldlr^−/−^* and *Apoe^−/−^*). These models provide valuable insight into early valvular dysfunction, but differ from human pathology in terms of timing, lesion stage, and hemodynamic environment. Notably, previous human studies have identified disease-driving VIC subsets in advanced calcified valves (59), yet such late-stage populations may be less amenable to therapeutic intervention. Third, the cell-cell communication analysis presented here relies on scRNA-seq expression data and lacks spatial context. As such, it may overlook spatial distribution and paracrine gradients that influence intercellular signaling *in situ*.

## Data Availability

The original contributions presented in the study are included in the article/Supplementary Material, further inquiries can be directed to the corresponding author/s.
