# Supplementary material for: Osteopontin-expressing valvular interstitial cell subpopulation as a driver of extracellular matrix remodeling in aortic valve disease
Source: Front Cardiovasc Med. 2026 Apr 7;13:1755830. doi: 10.3389/fcvm.2026.1755830 (PMC13095520; doi:10.3389/fcvm.2026.1755830)
Supplement: Supplementary file 1 [file Datasheet1.docx]

**Osteopontin-expressing valvular interstitial cell subpopulation as a driver of extracellular matrix remodeling in aortic valve disease**

Razvan D. Macarie, Monica M. Țucureanu, Letiția Ciortan, Mihai Bogdan Preda, Ileana Mânduțeanu and Elena Butoi

Inflammation Department, Institute of Cellular Biology and Pathology “Nicolae Simionescu”, Bucharest, Romania

**Corresponding author:*

Razvan D. Macarie – razvan.macarie@icbp.ro

**Supplementary material**

1. **Supplementary method**:

Trajectory inference was performed using the R package **Monocle3** (version 1.3.1) on VIC and VEC subsets. To preserve the previously defined cellular structure, the scVI latent space obtained during Seurat integration was used as the input for dimensionality reduction. UMAP embedding was computed from the scVI space using a minimum distance of 0.3 and 30 neighbors). Pseudotime ordering was performed by selecting VIC Meox1⁺ cells as the root population. Expression of selected VIC Spp1 cluster markers and ECM related genes (Fn1, Fmod, Spp1, Vim) and endothelial markers (Pecam1, Cdh5, Tek, Vwf) was visualized along the learned trajectories.

1. **Supplementary Figures**:

**Supplementary Figure 1**


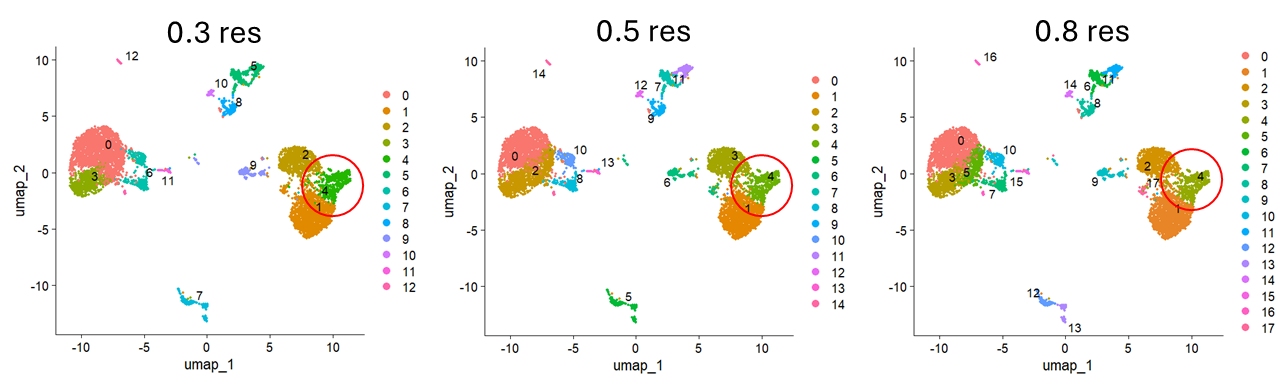


**Supplementary Figure 1** - VIC subclustering at 0.3, 0.5 and 0.8 resolution reveals a consistent VIC Spp1⁺ subpopulation

**Supplementary Figure 2**


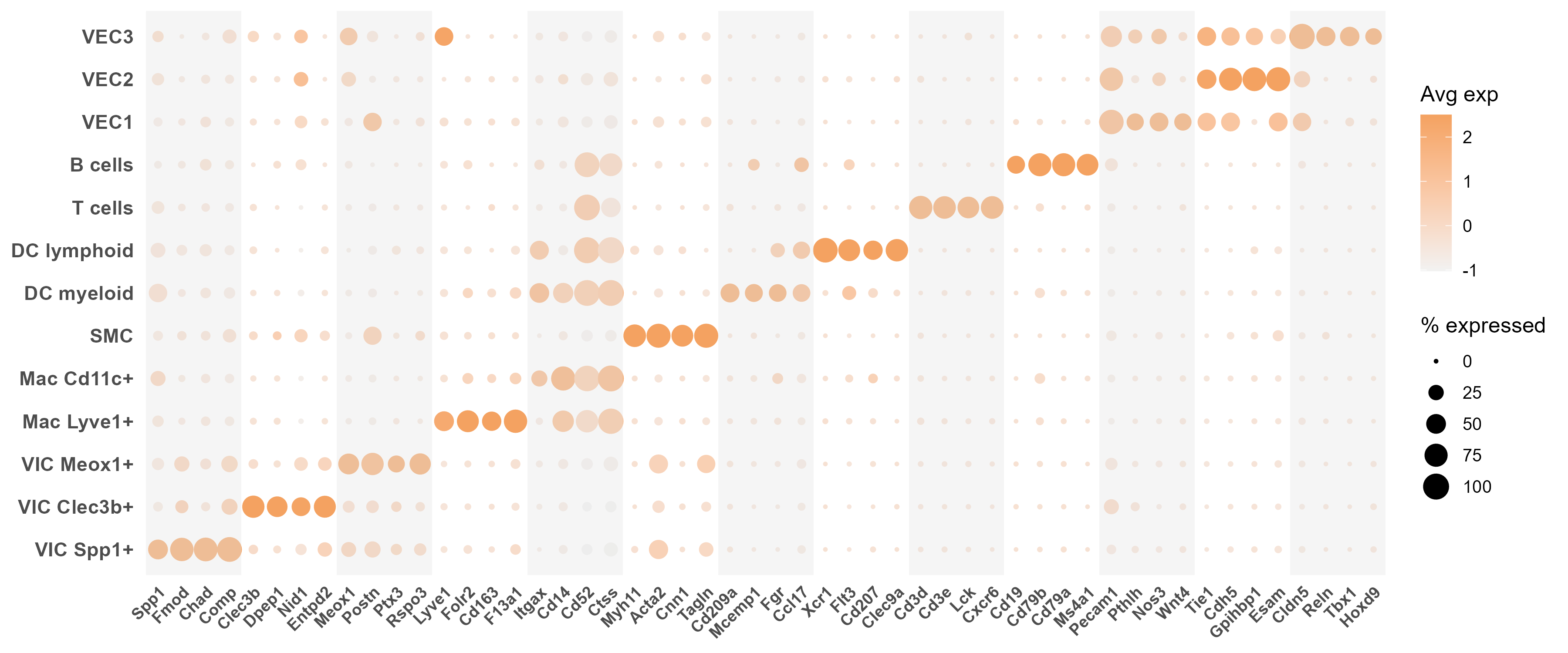


**Supplementary Figure 2** - Gene expression of key markers for the 13 cell clusters identified in scRNA-seq dataset.

**
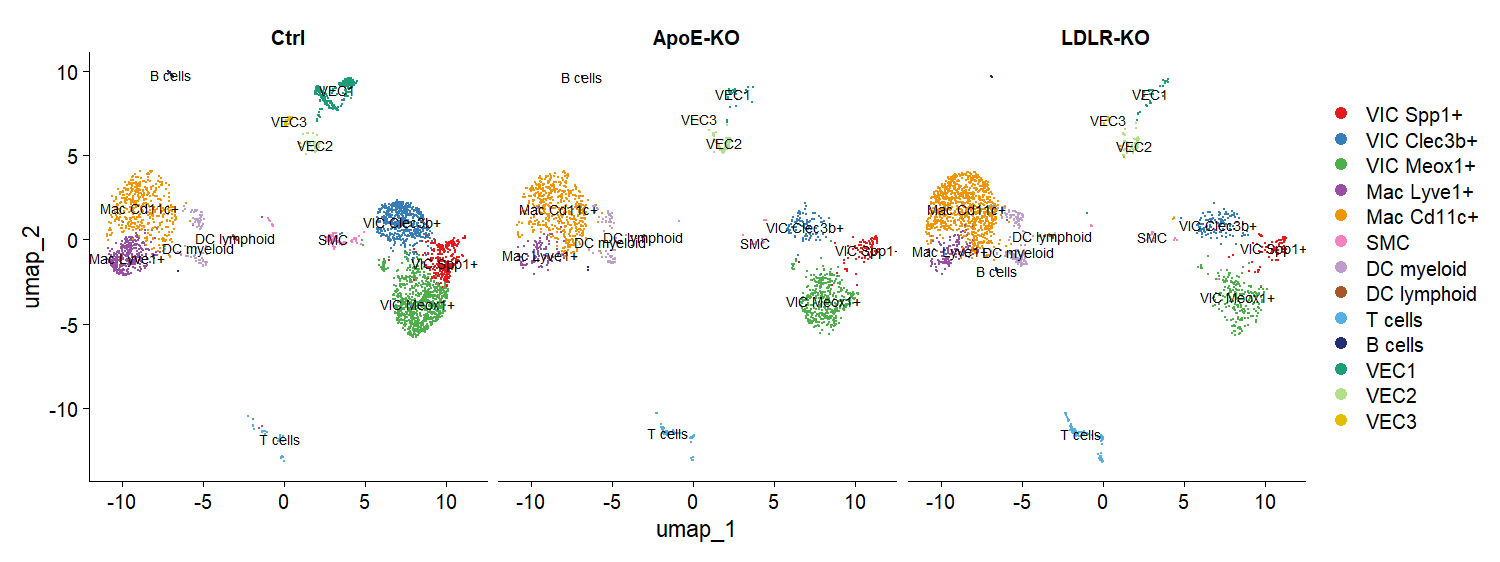
Supplementary Figure 3 -** a) UMAP visualization of integrated single-cell transcriptomes from mouse aortic valves, split by experimental condition (Ctrl, Ldlr^−/−^, and Apoe^−/−^). The VIC Spp1⁺ population shows variable representation across conditions, with a higher abundance in control samples compared with Apoe^−/−^ mice.


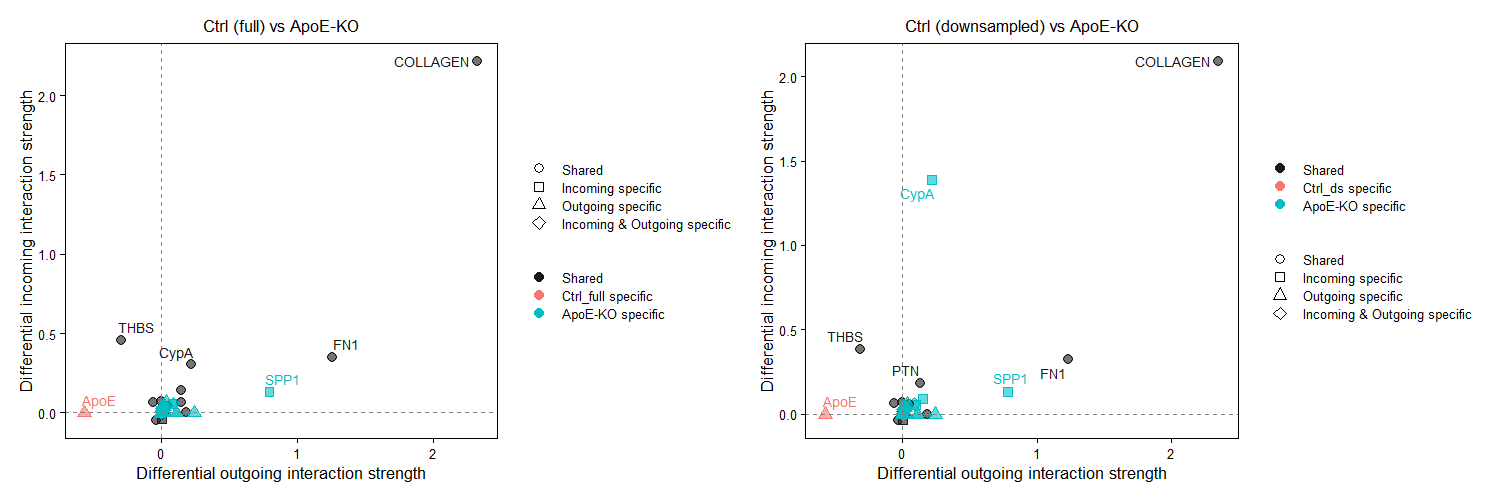

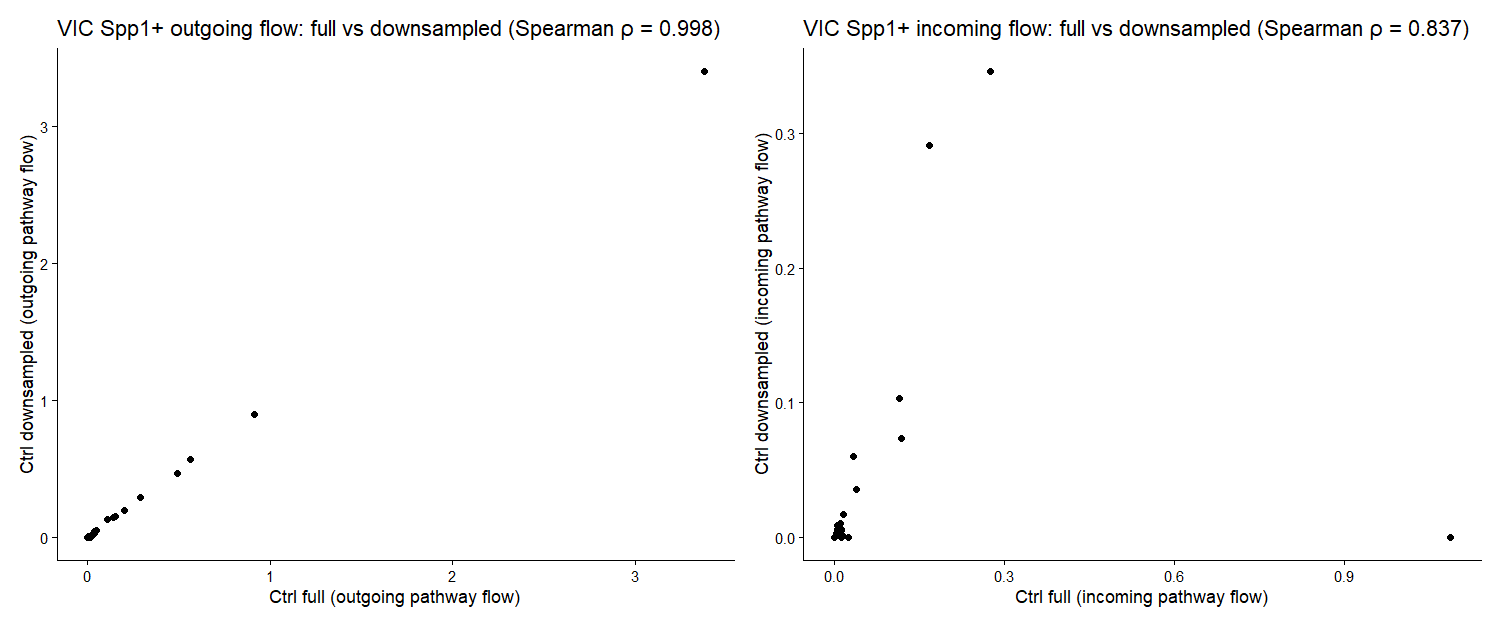


b

a

**Supplementary Figure 3**

b) Validation of CellChat signaling inference after downsampling. Ctrl VIC Spp1⁺ cells were randomly subsampled to match the number observed in Apoe^−/−^ mice, followed by a re-analysis of intercellular communication using CellChat. Scatter plots show the correlation between signaling interaction strengths inferred from the full Ctrl dataset and the downsampled dataset. Outgoing signaling patterns show near-perfect concordance (Spearman r ≈ 0.998), while incoming signaling also shows strong agreement (Spearman r ≈ 0.837), indicating that the inferred signaling networks are not driven by differences in cell abundance.

**Supplementary Figure 4**

**
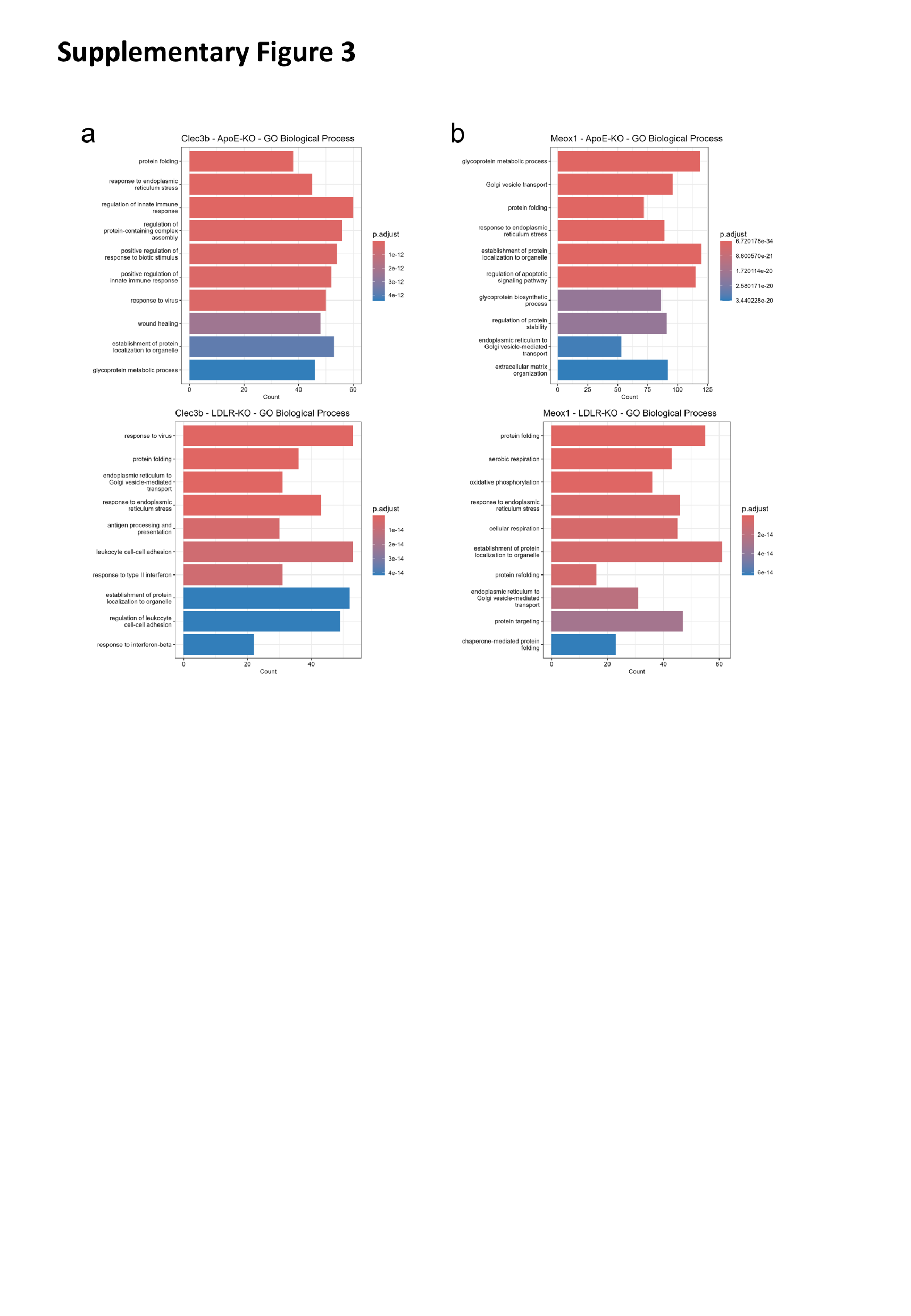
**

**Supplementary Figure 4** - GO enrichment analysis of DEGs in VIC Clec3b (a) and Meox1 (b) cells indicates involvement in protein folding and stability, hinting at an endoplasmic reticulum stress response in these VIC subpopulations

**
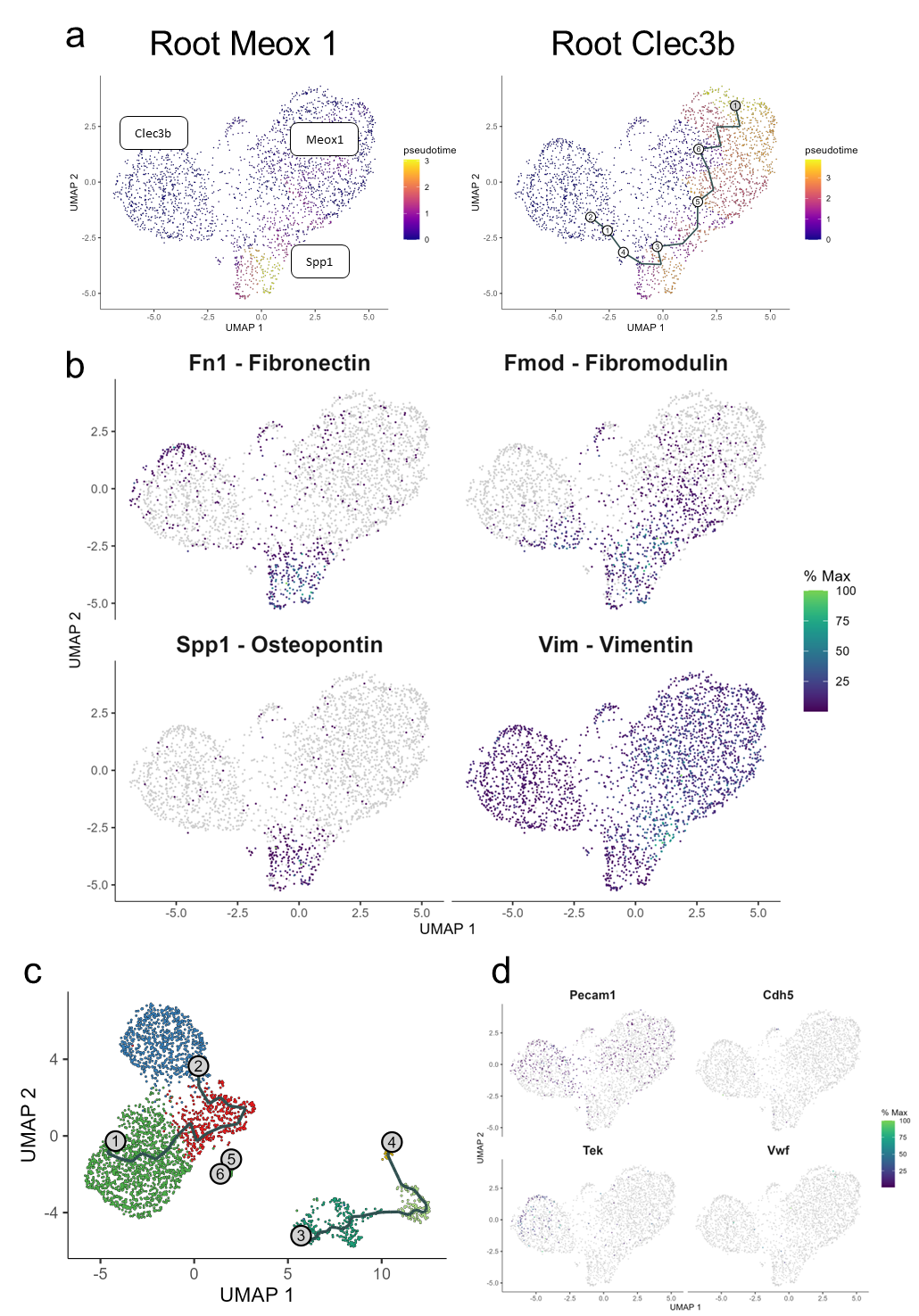
Supplementary Figure 5**

**Supplementary Figure 5 -** Trajectory inference suggests distinct VIC and VEC lineages and an intra-VIC progression toward the VIC Spp1⁺ state.

(a) VIC-only Monocle3 trajectory inference. Cells are colored by pseudotime after ordering with VIC Meox1⁺ cells specified as the root population. This analysis yields a continuous path along the VIC manifold in which later pseudotime becomes enriched toward the VIC Spp1⁺ region. Considering a root starting from the VIC Clec3b⁺ we see a different pattern with 2 points of pseudotime enrichment towards both VIC Meox1⁺ and VIC Spp1⁺.

(b) Expression of representative VIC Spp1⁺ associated and ECM genes (Fn1, Fmod, Spp1, Vim) overlaid on the ordered VIC trajectory. These markers exhibit spatial structure along the inferred trajectory, supporting the interpretation that the VIC Spp1⁺ state corresponds to a distinct transcriptional program associated with matrix remodeling/activation within the VIC compartment.

(c) Monocle3 trajectory graph learned on a joint embedding of valvular interstitial cells (VICs) and valvular endothelial cells (VECs). To preserve the transcriptional structure used throughout the study, monocle3 was run using the previously computed scVI space from Seurat (reducedDims “PCA” injected with scVI embeddings) and a UMAP computed from this latent space. Cells are colored by annotated cell identity (VIC Spp1⁺, VIC Clec3b⁺, VIC Meox1⁺, VEC1-3), and the principal graph/branch points inferred by monocle3 are overlaid. In this representation, VIC and VEC populations form independent trajectory graphs and no prominent bridging continuum between endothelial and interstitial lineages.

(d) Feature overlays for canonical endothelial markers (Pecam1, Cdh5, Tek, Vwf) projected onto the VIC embedding to assess evidence for endothelial-like transcriptional states within VIC clusters. Across the VIC, these endothelial markers remain largely restricted to the VIC Clec3b⁺ and VIC Meox1⁺, and do not display a progressive gradient toward VIC states, consistent with limited evidence for a broad endothelial-to-interstitial transitional continuum in this dataset.

**Supplementary Figure 6**


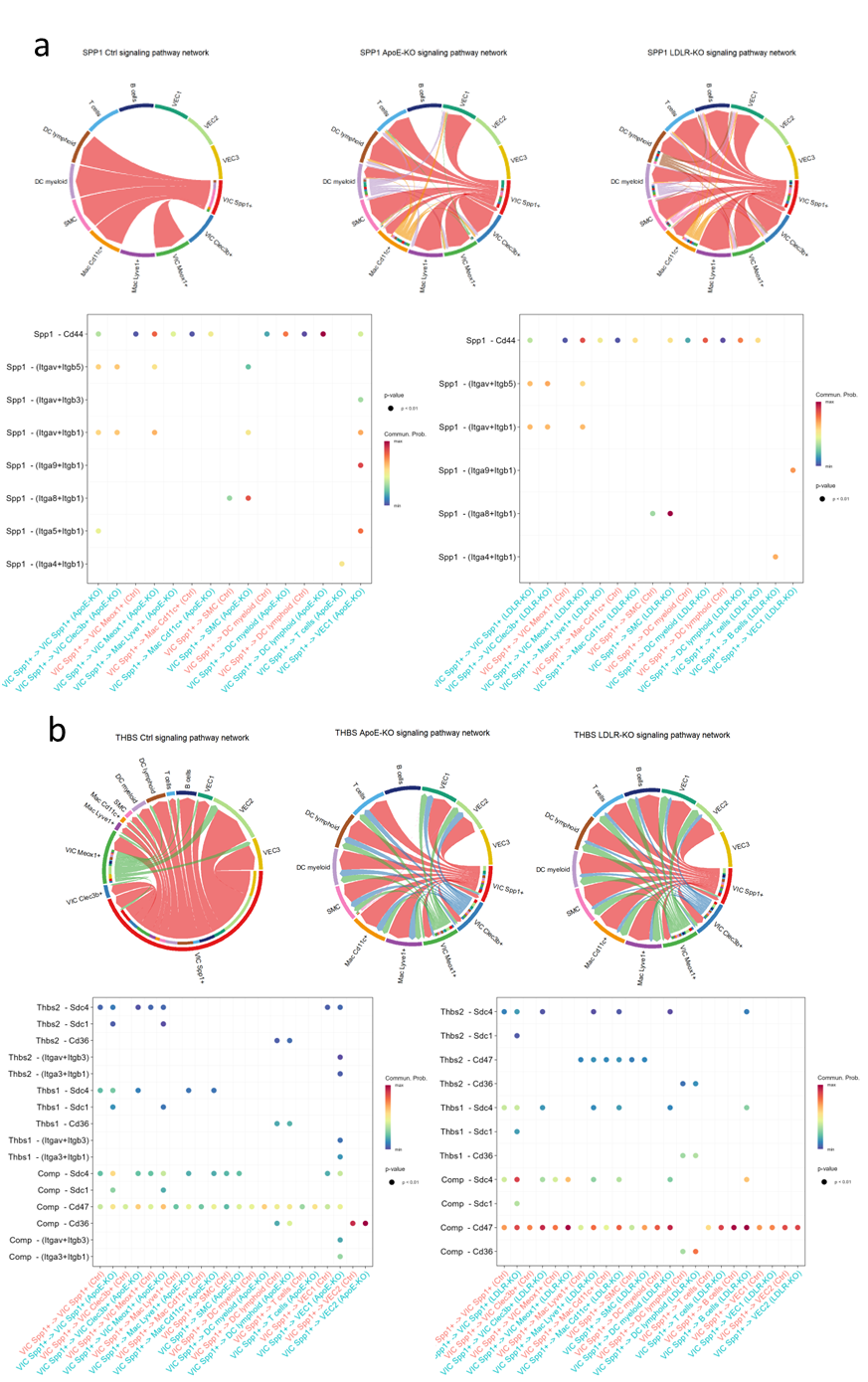


**Supplementary Figure 6 –** Predicted intercellular communication through Spp1 (a) and THBS1 (b) signaling pathways. Osteopontin (Spp1) secreted by VIC Spp1⁺ cells is predicted to interact with Cd44 expressed on VIC Meox1⁺, smooth muscle cells (SMCs), macrophages (Cd11c⁺), and dendritic cells. THBS1 signaling primarily occurs via COMP–CD47 and COMP–α3β6 ligand–receptor pairs.
